# SIRT6 participates in the quality control of aged oocytes via modulating telomere function

**DOI:** 10.18632/aging.101885

**Published:** 2019-03-29

**Authors:** Juan Ge, Congyang Li, Chunling Li, Zhenyue Huang, Juan Zeng, Longsen Han, Qiang Wang

**Affiliations:** 1 State Key Laboratory of Reproductive Medicine, Nanjing Medical University, Nanjing, China; 2 Center for Global Health, School of Public Health, Nanjing Medical University, Nanjing, China

**Keywords:** aging, sirtuin, oocyte quality, telomere, embryo development

## Abstract

It has been well recognized that oocyte quality declines in aging animals. However, to date, the underlying mechanism remains to be explored. In the present study, we report that oocytes and embryos from aged mice (42-45 weeks old) display the reduced expression of SIRT6 protein, accompanying with telomere shortening and DNA lesions. Moreover, we demonstrate that specific depletion of SIRT6 in oocytes induces dysfunctional telomeres and apoptosis of the resultant early embryos, leading to the developmental delay and cytoplasmic fragmentation. Importantly, we further find that overexpression of SIRT6 in aged oocytes promotes the telomere elongation in 2-cell embryos and lowers the incidence of apoptotic blastomeres. In summary, our data indicate a role for SIRT6 in modulating telomere function during oocyte maturation and embryonic development, and discover that SIRT6 reduction is an important point connecting maternal aging and quality control of oocyte/embryos.

## INTRODUCTION

Oocyte quality, an indicator of female reproductive health, is essential for embryo development and pregnancy outcome. Following the completion of nuclear and cytoplasmic maturation, fully-grown oocytes acquire the ability to fertilize and support embryo development [1]. Maternal age-related decline in fecundity is largely dependent on oocyte quality [2, 3]. Studies of women of advanced reproductive age revealed the mitochondrial dysfunction, spindle disorganization, and chromosome mis-segregation in oocytes [4]. These defects during meiosis sharply increase the incidence of infertility, miscarriage, and congenital malformation.

Sirtuins (SIRT1–7) have been implicated in influencing diverse biological events, such as aging, energy control, transcription, and apoptosis [5, 6]. For example, SIRT1 was initially discovered to deacetylate p53 tumor suppressor protein and function in chromatin regulation and genome stabilization [7]. SIRT2 was found to be associated with mitotic structures and to ensure normal cell division [8]. SIRT3 has been shown to regulate mitochondrial fatty acid oxidation by controlling the acetylation level of long-chain acyl-CoA dehydrogenase (LCAD) in mouse liver [9]. SIRT4 was identified as a lipoamidase that inhibits pyruvate dehydrogenase complex [10]. SIRT6 was initially characterized as a nuclear ADP-ribosyltransferase [11]. In mice, deficiency for SIRT6 results in genomic instability and metabolic defects, highlighting its importance in aging, energy supply and cancer [12]. Recently, we found that SIRT6 depletion leads to the aberrant spindles and chromosome congression failure in mouse oocytes [13]. It has also been reported that SIRT6 functions in the maintenance of telomeric chromatin structure and genomic integrity via the deacetylation of histone H3 lysine9 (H3K9) [14].

Telomeres are composed of repetitive G-rich sequences and associated proteins. It has been well recognized that telomeres serve multiple functions in protecting the ends of chromosomes from degradation and preventing illegitimate chromosomal recombination [15, 16]. Telomerase, also called terminal transferase, is a DNA polymerase that synthesizes telomeric DNA sequences [17]. In most somatic cell types, telomeres progressively shorten because of the absence of telomerase and the incomplete replication of the linear DNA molecules [18]. Maternal aging adversely impacts telomerase activity and telomere length in ovaries, likely through the long-term oxidative stress and activation of DNA damage checkpoint [19].

Although multiple pathways have been proposed to contribute to the decreased quality of aged oocytes, the underlying mechanisms are still lacking. Herein, our study identified a reduction of SIRT6 expression in aged oocytes. We further revealed that specific depletion of SIRT6 in mouse oocyte could induce the telomere dysfunction and compromises the developmental potential.

## RESULTS

### Reduced SIRT6 expression in oocytes and embryos from aged mice

**Figure 1:**
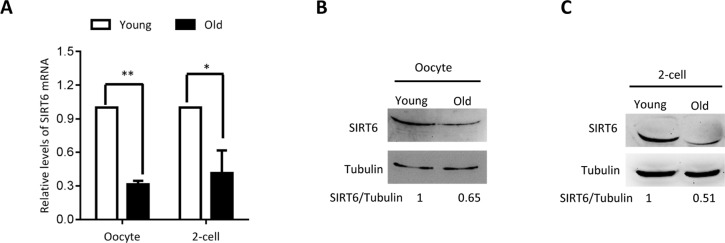
**SIRT6 expression in aged mouse oocytes and embryos**. Fully-grown oocytes and two-cell embryos from young and aged mouse were collected to evaluate SIRT6 expression. (**A**) Quantitative RT-PCR showing the lowered SIRT6 mRNA levels in aged oocytes and two-cell embryos. (**B**–**C**) SIRT6 protein expression in aged oocytes and two-cell embryos was verified by immunoblotting. Tubulin served as a loading control. Band intensity was calculated using ImageJ software, and the ratio of SIRT6/tubulin expression was normalized and values are indicated. Data are expressed as mean percentage ± SD of three independent experiments. *P <0.05, **P <0.01.

To dissect the potential relationship between SIRT6 and quality control of oocyte aging, we first examined the expression of SIRT6 in oocytes from old mice (42-45 weeks old). Analysis of quantitative real-time PCR showed that the relative mRNA levels of Sirt6 were significantly downregulated in aged oocytes in comparison to the oocytes from young mice (Figure 1A). In line with this, about a 60% of reduction in SIRT6 protein expression was detected in old oocytes (Figure 1B). Moreover, we found that the expression of SIRT6 protein was also dramatically lowered in 2-cell embryos isolated from old mice relative to controls (Figure 1C). These findings indicate that SIRT6 reduction might contribute to the compromised quality of aged mice.

### Telomere dysfunction and DNA lesion in aged oocytes and embryos

It has been widely reported that aging is associated with shorter telomere function in diverse cell types and tissues. Therefore, we decided to evaluate the status of telomere in oocytes/embryos from old mice. Telomeres protect the ends of the chromosome from fusion to other chromosomes during cell division. Chromosomal rearrangements due to telomere shortening in cancer cells are thought to result from the extensive chromosome fusion [20].

**Figure 2:**
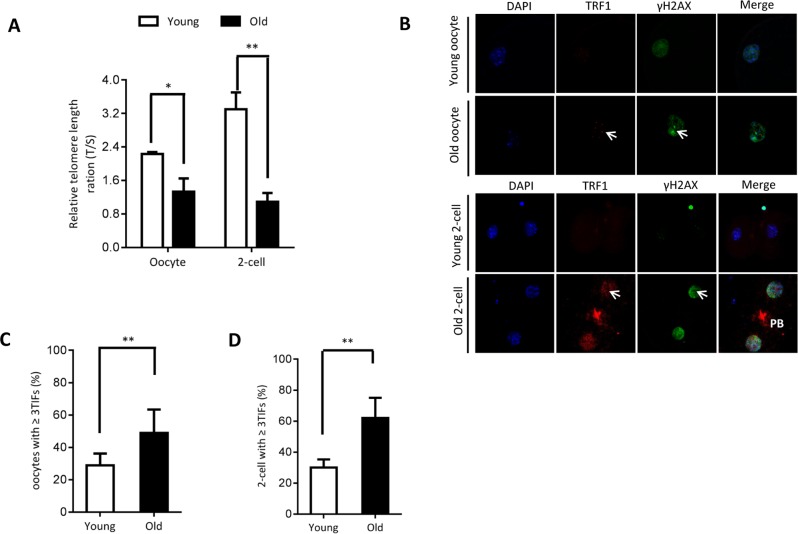
**Telomere dysfunction and DNA damage in aged oocytes and embryos.** (**A**) Relative telomere length in oocytes and two-cell embryos is expressed as a T/S ratio determined by quantitative RT-PCR analysis. Data are expressed as mean percentage ± SD of three independent experiments (oocytes: n=114 young, n=106 old; embryos: n=85 young, n=86 old). (**B**) Representative images of oocytes/two-cell embryos stained with antibodies against TRF1 (red) and γH2AX (green), and co-stained with Hoechst 33342 for chromosomes (blue). PB, polar body. Scale bars, 25 µm. (**C**–**D**) Quantification of DNA damage-induced foci (TIFs) from (B). TIFs were detected by co-localization of TRF1 and γ-H2AX, and cells with at least 3 TIFs were scored. Data represent averages of 3–10 fields. Error bars indicate SD (oocytes: n=24 young, n=26 old; embryos: n=35 young, n=38 old). Statistical analyses were performed with Student’s t-test. *P <0.05, **P <0.01.

As shown in Figure 2A, we found that the relative telomere lengths were markedly decreased in both oocytes and 2-cell embryos from old mice. Telomeric repeat-binding factor 1 (TRF1) is a homodimeric protein, which interacts with the telomeric DNA at its C-terminus [21]. TRF1 blocks telomerase access to the telomeres, and overexpression of TRF1 induces a gradual decline in telomere length [22, 23]. Replication stress induces telomere dysfunction, which can activate the DNA damage response factors, forming the so-called telomere dysfunction-induced foci (TIFs) [24]. Next cells were double-stained for phosphorylated H2AX (γH2AX, a surrogate marker for DNA damage) and TRF1. As shown in Figure 2B–2D, the numbers of TIFs are significantly increased in old oocytes and embryos when compared to young cells (arrows). This indicates that the elevated DNA lesions and telomere dysfunction in aged oocytes and embryos. Accumulation of unrepaired DNA damage generates signals that affect genome integrity and cell cycle progression.

### Effects of SIRT6 depletion in oocyte on embryo development

**Figure 3:**
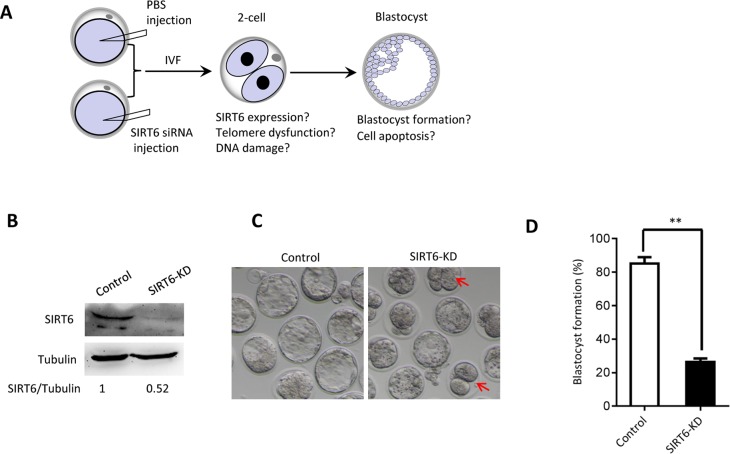
**SIRT6 knockdown in oocytes results in the developmental delay of early embryos.** (**A**) Schematic presentation of the SIRT6-knockdown experiments. (**B**) Efficiency of SIRT6 knockdown (SIRT6-KD) after siRNA injection was verified by immunoblotting. Tubulin served as a loading control. Band intensity was calculated using Image J software. (**C**) Representative contrast images of blastocyst embryos derived from control and SIRT6-KD oocytes. Red arrows indicate the developmental delay. (**D**) The percentage of zygotes that successfully progressed to the blastocyst stage during *in vitro* culture (n= 65 for control; n=55 for SIRT6-KD). Data are expressed as mean percentage ± SD of three independent experiments. Statistical analyses were performed with Student’s t-test. **P <0.01.

**Figure 4:**
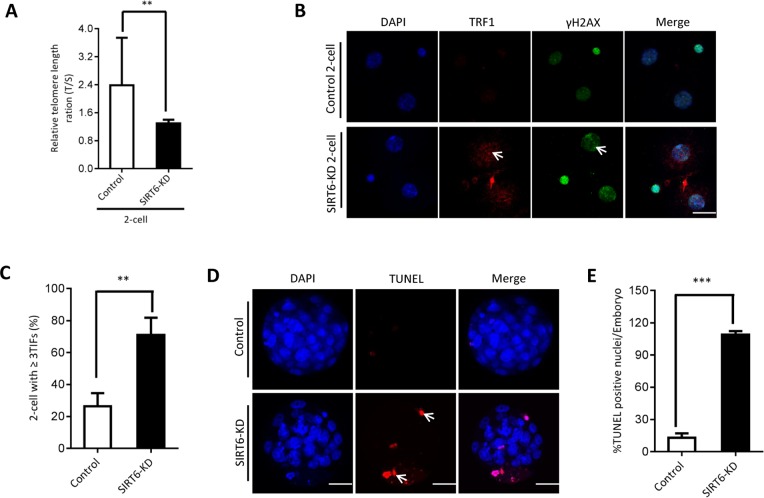
**SIRT6 knockdown in oocytes induces DNA damage and apoptosis of early embryos.** (**A**) Relative telomere length in two-cell embryos is expressed as a T/S ratio determined by quantitative RT-PCR analysis. Data are expressed as mean percentage ± SD of three independent experiments (n=125 for control; n=128 for SIRT6-KD). (**B**) Representative images of young and SIRT6-KD two-cell embryos stained with antibodies against TRF1 (red) and γH2AX (green), and co-stained with Hoechst 33342 for chromosomes (blue). Scale bars, 25 µm. (**C**) Quantification of DNA damage-induced foci (TIFs) from (B). TIFs were detected by co-localization of TRF1 and γ-H2AX, and cells with at least 3 TIFs were scored (n=35 for control; n=32 for SIRT6-KD). Data represent averages of 3–10 fields. (**D**) TUNEL analysis of control and SIRT6-KD embryos. Embryos were labeled with Hoechst 33342 (blue) for DNA and by TUNEL for fragmented DNA (red). Arrowheads point to the apoptotic cells in blastocysts. (**E**) Quantification of control and SIRT6-KD blastocysts with TUNEL positive nuclei (n=82 for control; n=75 for SIRT6-KD). Statistical analyses were performed with Student’s t-test. *P<0.05, ** P<0.01, ***P<0.001.

The phenotypes in old oocytes/embryos mentioned above prompted us to ask whether they are associated with the loss of SIRT6 in oocytes. To address this question, normal MII oocytes were microinjected with specifically designed siRNAs to knock down the SIRT6 level (SIRT6-KD, Figure 3A). Immunoblotting confirmed that siRNA injection led to a dramatic reduction in SIRT6 protein expression in oocytes (Figure 3B). Next, we carried out *in vitro* fertilization (IVF) of control and SIRT6-KD oocytes, and then zygotes were cultured to check the subsequent embryonic development. As shown in Figure 3C–3D, early embryos derived from SIRT6-KD oocytes displayed a significant decrease in blastocyst formation (red arrows) and increase in cytoplasmic fragmentation in comparison to controls. These observations indicate the compromised developmental potential of early embryos when SIRT6 was abated in oocytes. SIRT6 loss is associated with the dysfunction of telomeres via affecting telomeric chromatin structure. In line with notion, telomeres shortened markedly in 2-cell embryos derived from SIRT6-KD oocytes compared to control embryos (Figure 4A). Moreover, we found that >3 TIFs were frequently detected in 2-cell embryos originated from SIRT6-KD oocytes (Figure 4B–4C). Telomeres are composed of repetitive G-rich sequence and specific binding proteins that together stabilize the ends of chromosomes. These structures are closely related to cellular apoptosis [25, 26]. To evaluate the apoptotic status, Terminal dUTP Nick End Labeling (TUNEL) assay was conducted on control and SIRT6-KD blastocysts. As shown in Figure 4D–4E, TUNEL positive nuclei were hardly detected in normal blastocyst. In contrast, the proportion of apoptotic blastomeres was significantly increased in embryos derived from SIRT6-KD oocytes. Altogether, these results indicate that oocyte SIRT6 is important for maintaining the telomere function and genome integrity in early embryos, thereupon determining the developmental competence.

### SIRT6 overexpression partially rescues defective phenotypes of embryos from aged mice

**Figure 5:**
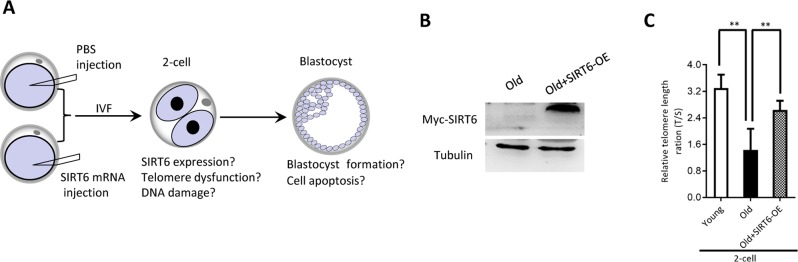
**SIRT6 overexpression in aged oocytes increases the telomere length in early embryos.** (**A**) Schematic presentation of the SIRT6 overexpression experiments. (**B**) Efficiency of SIRT6 overexpression (SIRT6-OE) after mRNA injection was verified by immunoblotting. (**C**) Relative telomere length expressed in two-cell embryos is expressed as a T/S ratio determined by quantitative RT-PCR analysis. Data are expressed as mean percentage ± SD of three independent experiments (n=86 for young; n=85 for old; n=80 for old+SIRT6-OE). **P <0.01.

**Figure 6:**
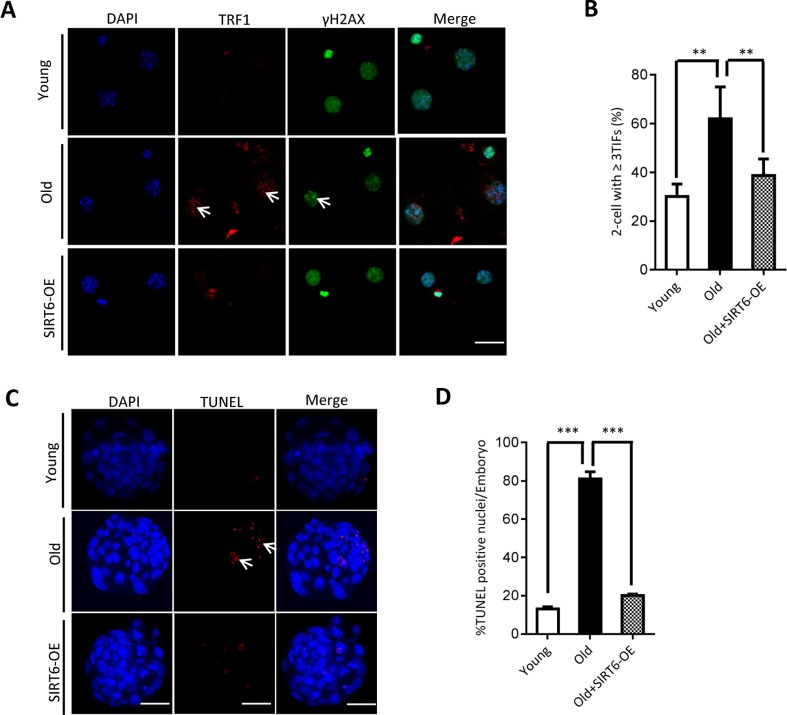
**SIRT6 overexpression in aged oocytes partly prevented the defective phenotype of early embryos.** (**A**) Representative images of young, old, and SIRT6-OE two-cell embryos stained with antibodies against TRF1 (red) and γH2AX (green), and co-stained with Hoechst 33342 for chromosomes (blue). Scale bars, 25 µm. (**B**) Quantification of DNA damage-induced foci (TIFs) from (A). TIFs were detected by co-localization of TRF1 and γ-H2AX, and cells with at least 3 TIFs were scored (n=26 for young; n=28 for old; n=25 for old+SIRT6-OE). (**C**) TUNEL analysis of young, old, and SIRT6-OE embryos. Embryos were labeled with Hoechst 33342 (blue) for DNA and by TUNEL for fragmented DNA (red). Arrowheads point to the apoptotic cells in blastocysts. Scale bars, 25 μm. (**D**) Quantification of young (n=90), old (n=82), and SIRT6-OE (n=78) blastocysts with TUNEL positive nuclei. Data are expressed as mean percentage ± SD from three independent experiments. ** P<0.01, ***P<0.001.

Our above findings suggest the potential connection between SIRT6 insufficiency in oocytes and telomere dysfunction in early embryos from old mice. Therefore, we next sought to determine whether elevating expression of SIRT6 in aged oocytes could alleviate the deficient telomere in early embryos. To do this, we conducted SIRT6 overexpression experiments (SIRT6- OE) followed by IVF (Figure 5A). Immunoblotting assay verified the ectopic expression of exogenous SIRT6 protein in oocytes (Figure 5B). The telomere status and apoptosis in the resulting two-cell embryos and blastocysts were then evaluated, respectively. As shown in Figure 5C, ectopic expression of SIRT6 in aged oocytes promoted the telomere elongation in the subsequent 2-cell embryos. In line with this, the frequency of TIFs occurred in old embryos overexpressing SIRT6 was remarkably lowered (Figure 6A–6B). Moreover, we noticed that forced overexpression of SIRT6 reduces the percentage of apoptotic blastomeres in old blastocyst (Figure 6C–6D). Collectively, these data indicates that loss of SIRT6 in aged oocytes is an important factor inducing the dysfunctional telomeres and developmental abnormalities in early embryos.

## DISCUSSION

Telomere is a region of repetitive nucleotide sequences at each end of a eukaryotic chromosome. Telomeres can not only prevent genetic information loss, but also protect the chromosomes from end-to-end fusions to keep genome stability [27, 28]. SIRT6 interacts with multiple chromatin-related proteins and possesses ADP-ribosylase and deacetylase activity [14, 29]. Depletion of SIRT6 has been reported to result in cellular senescence because of the telomere dysfunction [14]. Silencing of a telomere-proximal gene also requires SIRT6 [30]. Moreover, to activate the downstream DDR factors and DNA repair, SIRT6 is recruited to DNA double-strand breaks (DSBs) following DNA damage [31, 32]. In this study, we found that SIRT6 knockdown in oocytes causes the reduction in telomere length in early embryos. Telomere-binding proteins TRF1 and TRF2 bind specifically to the double-stranded TTAGGG repeats, and are important for the control of telomeric length and structure [33]. TRF1 has been reported to play a key role in both telomerase-dependent telomere maintenance and alternative lengthening [34, 35]. In support of this conception, our data showed that the frequency of TIFs was dramatically increased in 2-cell embryos when SIRT6 was knocked down in oocytes (Figure 4).

Consequently, the SIRT6-depleted embryos fragmentation, with the apoptotic blastomeres (Figure 3). Altogether, these findings strongly suggest that SIRT6 is an essential factor determining the telomere structure and genomic integrity during mouse early embryogenesis. It has been reported that SIRT6 can modulate the acetylation level of H3K56 and H3K9 in mitotic cells [14, 36]. Therefore, telomere dysfunction in SIRT6-KD oocytes is likely correlated with the altered telomere chromatin structure induced by elevated acetylation. Our ongoing research is to address this question.

Presence of telomerase activity in female germ cells ensures the proper meiotic process and the generation of euploid eggs [37]. It has been shown that DNA damage and telomere shortening resulted from the overproduction of free radicals promote ovarian aging [38]. Decreased Tert expression and telomerase activity have been detected in oocytes from reproductively aged females [39]. Similarly, here we notice the reduced telomere length and increased DNA lesions in oocytes and 2-cell embryos from aged mice (Figure 2). Telomere shortening compromises the chromosome synapsis and meiotic apparatus in germ cells, acting to produce aneuploid embryos after fertilization [40, 41]. Most aneuploidies in eggs arise in frequency with advancing maternal age [3, 42]. It is worthy of noting that SIRT6 expression was also remarkably decreased in these old oocytes (Figure 1). We have previously revealed that SIRT6 is critical for the proper assembly of meiotic structure during mouse oocyte meiosis [13]. Importantly, overexpression of SIRT6 in aged oocytes could partly prevent the telomere dysfunction and apoptosis in the subsequent early embryos (Figure 6). Consistent with this observation, telomere shortening was reported to be involved in the cytoplasmic fragmentation of preimplantation embryos [43].

In conclusion, our results suggest that SIRT6-controlled telomere structure is a crucial factor controlling oocyte quality from aged mice. Meanwhile, we propose that maternal aging results in the SIRT6 insufficiency in oocytes, which in turn disrupts the telomere function and genomic integrity, and consequently, contributing to the compromised developmental potential of the resultant early embryos.

## MATERIALS AND METHODS

All chemicals and culture media were purchased from Sigma (St. Louis, MO, USA) unless otherwise stated.

### Mice

All animal experiments followed the rules of the Animal Care and Use Committee of Nanjing Medical University. ICR young female mice (3-4 week old) were used in our experiments. 42-45 weeks old female mice (near the end of their reproductive lifespan) were used as a natural aging mouse model.

### Antibodies

The following antibodies were purchased: rabbit polyclonal anti-SIRT6 antibody (Sigma, Cat#: S4197), rabbit polyclonal anti-TRF1 antibody (Abcam, Cat#: ab1423), mouse monoclonal anti-γH2AX antibody (Abcam, Cat#: ab22551), mouse monoclonal anti-Myc antibody (Abcam, Cat#: ab18185).

### Oocyte collection

To obtain MII oocytes, female mice were superovulated by injecting 5U of pregnant mare’s serum gonadotropin (PMSG) followed by 5U of human chorionic gonadotropin (hCG) 48h after PMSG priming. Mice were euthanized by cervical dislocation 14h after the hCG injection. Cumulus–oocyte complexes (COCs) were isolated from oviduct ampullae, and denuded MII oocytes were then obtained by removing the cumulus mass in medium containing 0.5mg/ml hyaluronidase at 37°C

### *In vitro* fertilization, embryo collection and culture

IVF assays were performed following our published protocols [44]. Sperm were collected from the dissected epididymis of ICR mice aged 10–20 weeks and left to capacitate for 1 hour in HTF fertilization medium (Millipore, Merck) supplemented with 10 mg/ml BSA. Then, dispersed spermatozoa were added to HTF drops containing COCs for fertilization. Zygotes were cultured into KSOM medium (Millipore, Merck) at 37 °C in a humidified atmosphere of 5% CO2, 5% O2 and 90% N2.

### Plasmid construction and mRNA synthesis

Total RNA was extracted from mouse oocytes/embryos using Arcturus Pico Pure RNA Isolation Kit (Applied Biosystems, CA, USA), and the cDNA was produced with QIA quick PCR Purification Kit (Qiagen, Germany). PCR products were cloned into the pCS2^+^ vector with Myc tags. mRNA synthesis was conducted by *in vitro* transcription with SP6 mMESSAGE mMACHINE (Ambion, CA) as we reported previously [45]. The related primer sequences can be found in Supplementary Table 1.

### Knockdown and overexpression analysis

Microinjection was performed employing an inverted microscope (Eclipse Ti-S, Nikon) equipped with a micromanipulator (Narishige) as we reported previously [46]. In knockdown experiments, SIRT6-specific siRNA was diluted to give a stock concentration of 1mM, and 5pl solution was injected into oocytes. In overexpression experiment, 10pl SIRT6 cRNA solution (10 ng/µl) was injected into the cytoplasm of MII-arrested oocytes. Oocytes were cultured for 3 hours before the subsequent manipulation. The siRNA sequences can be found in Supplementary Table 1.

### Western blotting

A total of 100 oocytes were lysed in Laemmli sample buffer with protease inhibitor, incubated at 95°C for 5 min, then stored at -20°C. Protein extracts were separated using SDS–PAGE and transferred to PVDF membranes. After blocking, the membranes were incubated at 4 °C overnight with anti-SIRT6 antibody (1:1,000), or anti-Myc antibody (1:1,000). After three washes, protein samples were incubated with horseradish peroxidase (HRP)-conjugated secondary antibodies (1:5,000; Thermo Fisher Scientific) at room temperature for 1 hour. The bands were visualized using an ECL Plus Western Blotting Detection System (GE Healthcare, Little Chalfont, UK).

### Quantitative real-time PCR

Quantitative RT–PCR was performed as we described previously [47]. Total cellular RNA was isolated from 50 oocytes/embryos using an Arcturus PicoPure RNA Isolation kit (Applied Biosystems) RNA queous-Micro Kit (Ambion, TX). GAPDH was used as an internal control. Average telomere length was measured from total genomic DNA using a RT-PCR assay according to the published protocol [48, 49]. Primer sequences are listed in Supplementary Table 1.

### Immunofluorescence

Oocytes/embryos were permeabilized with 0.5% Triton for 4-5 min, washed in PBS containing 0.1% polyvinyl pyrrolidone (PBS–PVP) for three times. Following a fixation step with 3.7% paraformaldehyde, oocytes or two-cell embryos were washed in PBS–PVP for 3 times and treated with blocking buffer (1% BSA-supplemented PBS) for 1 hour. Samples were incubated overnight at 4°C with rabbit polyclonal anti-TRF1 antibody (1:300) and mouse monoclonal anti-γH2AX antibody (1:200). After multiple washes, cells were incubated with Alexa Fluor 555–conjugated goat anti-rabbit and Alexa Fluor 488–conjugated goat anti-mouse secondary antibodies (1:150; Molecular Probes) for 1 hour at room temperature. Chromosomes were counterstained with Hoechst 33342 (1:250) for 10 min. Samples were mounted on slides with a drop of antifade medium (Vectashield, Vector Laboratories, CA, USA) and then examined under a laser scanning confocal microscope (LSM 710; Carl Zeiss, Oberkochen, Germany). For each antibody used, immunofluorescence was performed on oocytes/embryos from young and old mice in parallel and identical conditions. Images were always acquired using the same confocal microscope settings. The amount of γH2AX foci was analyzed using ImageJ (NIH, USA). To evaluate apoptosis, fixed embryos were processed for TUNEL analysis (In Situ Cell Death Detection Kit, Fluorescein, Roche).

### Statistical analysis

All experiments were repeated at least three times. Data are presented as mean ± SD, unless otherwise indicated. Statistical comparisons were made with Student’s test and ANOVA when appropriate using Prism 5 software (GraphPad, San Diego, CA, USA). P<0.05 was considered to be significant.

## Supplementary Material

Supplementary Table
