# Supplementary material for: SIRT6 participates in the quality control of aged oocytes via modulating telomere function
Source: Aging (Albany NY). 2019 Mar 29;11(7):1965–76. doi: 10.18632/aging.101885 (PMC6503879; doi:10.18632/aging.101885)
Supplement: Supplementary Table [file aging-11-101885-s001.pdf]

SUPPLEMENTARY MATERIAL

Supplementary Table 1. Primer sequences of genes for SiRNA.

Gene	***Primer sequence***
Sirt6	F: 5' –GCAGUGCAUGUUUCGUAUATT– 3'
	R: 5' –UAUACGAAACAUGCACUGCTT– 3'
**Primer sequences of gene for cloning**	
Gene	***Primer sequence***
Sirt6	F: 5' –GGCCGGCCATGTGGCAGTCCTCCAGCGTG– 3'
	R: 5' –GGCGCGCCTCAGCTGGGGGCAGCCTC – 3'
**Primer sequences of genes for qRT-PCR**	
Gene	***Primer sequence***
GAPDH	F: 5' –CTTTGTCAAGCTCATTTCCTGG – 3'
	R: 5' –TCTTGCTCAGTGTCCTTGC – 3'
Sirt6	F: 5' –ATGTCGGTGAATTATGCAGCA– 3'
	R: 5' –GCTGGAGGACTGCCACATTA– 3'
36B4	F: 5'–ACTGGTCTAGGACCCGAGAAG– 3'
	R: 5' –TCAATGGTGCCTCTGGAGATT– 3'
Telomere	F: 5' –CGGTTTGTTTGGGTTTGGGTTTGGGTTTGGGTTTGGGTT– 3'
	R: 5' –GGCTTGCCTTACCCTTACCCTTACCCTTACCCTTACCCT– 3'
